# Childhood cancer registration in Britain: capture-recapture estimates of completeness of ascertainment

**DOI:** 10.1038/bjc.2011.70

**Published:** 2011-03-15

**Authors:** M E Kroll, M F G Murphy, L M Carpenter, C A Stiller

**Affiliations:** 1 Childhood Cancer Research Group, University of Oxford, Richards Building, Oxford OX3 7LG, UK; 2 Cancer Epidemiology Unit, University of Oxford, Richard Doll Building, Oxford, OX3 7LF, UK; 3 Department of Public Health, University of Oxford, Rosemary Rue Building, Oxford OX3 7LF, UK; 4 Nuffield College, University of Oxford, New Road, Oxford OX1 1NF, UK

**Keywords:** childhood cancer, childhood leukaemia, registration, completeness, capture-recapture

## Abstract

**Background:**

Completeness of ascertainment is a very important aspect of cancer registration. There is no recent published estimate for childhood cancer in Britain.

**Methods:**

We estimated completeness of ascertainment by the National Registry of Childhood Tumours for cancer diagnosed under age 15 years in residents of Britain during 2003–04. Stratified two-source capture-recapture was applied to notifications from general cancer registries (CRs) and specialist clinicians. Variation in notification patterns was assessed by logistic regression. Results were verified by cross-checking with Hospital Episode Statistics for leukaemia patients from England born in 1998 and diagnosed before 2005.

**Results:**

CRs notified 92–96% of registrations, and specialist clinicians 93%. Notification patterns varied slightly according to registry region, age at diagnosis, diagnostic group, socioeconomic status, and whether the patient had died. Irrespective of stratification by these factors, the overall completeness estimate was 99–100% (assuming independence of sources). Estimated completeness was at least 99% within all subgroups, except for one region (Thames 98–99%) and two small diagnostic groups (germ-cell and gonadal cancer 98–99%, melanoma and non-skin cancer 97–98%).

**Interpretation:**

The independence assumption cannot be fully justified, as both sources used records from treatment centres. With this caveat, ascertainment of recently diagnosed childhood cancer in Britain appears to be virtually complete.

In calculating cancer incidence and survival rates from population-based cancer registry data, completeness of ascertainment of cases is very important. In Britain there is a special registration process for cancer in children, which may differ in completeness from the equivalent process in adults.

Cancer registry completeness can be evaluated by independent case ascertainment, capture-recapture, or death-certificate methods (Parkin and Bray, 2009). Methods involving death certificates are difficult to apply to childhood cancer, because the proportion of patients who die within a few years of diagnosis is much lower than for adults. A simple form of capture-recapture, stratifying for factors that may affect the probability of notification (Hook and Regal, 1995), is appropriate for the National Registry of Childhood Tumours (NRCT), as there are two principal sources of notification. The national Hospital Episode Statistics (HES) database provides a separate list of cases for cross-checking.

Two-source capture-recapture was previously used to estimate completeness of ascertainment by the NRCT for childhood leukaemia and non-Hodgkin lymphoma diagnosed during 1966–1983 (Draper *et al*, 1991). We are not aware of any other published estimate of completeness of national cancer registration in Britain specifically for children. Capture-recapture has been used to estimate completeness of ascertainment of childhood cancers in New Zealand for the diagnosis period 1990–1993 (Dockerty *et al*, 1997) and of childhood acute leukaemia in three cities in Brazil during 2001 (Azevedo-Silva *et al*, 2009).

A comprehensive literature review reported that records-based studies of childhood leukaemia incidence generally found a positive association with higher socioeconomic status (SES) (Poole *et al*, 2006). More recently, for the lymphoid subgroup in particular (the majority of childhood leukaemia in developed countries, peaking at ages 2–3 years) a similar association has been reported for children in the USA (Adelman *et al*, 2007) and England and Wales (Stiller *et al*, 2008), at ages 0–4 and 1–4 years respectively. One possible explanation is that ascertainment might be less complete for children of lower SES. It has also been suggested that, for cancer at any age, completeness may vary between registry regions of England and Wales (Quinn *et al*, 2001).

In this paper we estimate recent completeness of ascertainment of childhood cancers in Britain, and investigate some factors that might affect the probability of notification: registry region of residence, age at diagnosis, diagnostic group, whether the patient had died, and (in England and Wales) SES. The results should aid the interpretation of population-based studies of childhood cancer in Britain.

## Materials and methods

### Materials

Cancer registration in the UK relies mainly on routine medical and administrative records from National Health Service (NHS) hospitals where cancer is diagnosed and treated, supplemented by notifications derived from death certificates mentioning cancer. The system operates through a network of general cancer registries (CRs), which has covered the whole geographical area of Britain (England, Wales and Scotland) since 1962. Currently there are 11 CR zones for the UK, comprising Northern Ireland, Scotland, Wales, and eight regions of England. The NRCT exists to give special attention to cases diagnosed under the age of 15 years.

The NRCT matches and amalgamates notifications from two principal sources: first the CRs, described above, and second a register of patients seen by clinicians affiliated to the Children's Cancer and Leukaemia Group (CCLG) (until 1 August 2006, the United Kingdom Children's Cancer Study Group (UKCCSG)), the organisation that co-ordinates paediatric oncology in the UK and Eire. The NRCT also receives copies of death certificates with neoplasm given as the underlying cause for deaths occurring in Britain under the age of 20 years. In the very rare event that a valid death-certificate notification is not ascertained through another source, the case is registered and the date of death is used in place of the date of diagnosis.

Registered patients for whom a death certificate has not been received within 3 years of diagnosis are submitted for tracing and flagging on the NHS Central Registers for England, Wales and Scotland, so that embarkation, death and subsequent primary cancer can be ascertained. Further information is obtained from specialist childhood cancer registries within three of the eight English regions (North West, Northern/Yorkshire and West Midlands) and, for leukaemia patients only, from registers of participants in national clinical trials supported by the Medical Research Council. For patients notified by the CCLG, the accuracy of the information held by the NRCT is verified and updated through a series of routine follow-up enquiries to the notifying clinicians. The diagnostic codes provided on death certificates and on pathology reports (included with most CCLG notifications) are checked against the description in words, by a medically qualified member of staff. The coding system is standardised and periodically updated. Currently, all cases are coded to the site and type codes of the third edition of the International Classification of Diseases for Oncology (ICD-O), and hence to the third edition of the International Classification of Childhood Cancer (ICCC3) (Steliarova-Foucher *et al*, 2005).

After processing, registrations are notified by the NRCT to the general cancer registration system: retrospectively in February 2006 for cases diagnosed during 1981–2002, and prospectively since February 2007 through quarterly data exchange with the CRs.

### Methods

#### Data definitions

We examined registration of cancer diagnosed between 1 January 2003 and 30 December 2004 in children aged under 15 years who were resident in England, Wales or Scotland. ‘Cancer’ was defined as in ICCC3, and included intracranial and intraspinal (CNS) tumours of benign and uncertain behaviour. We excluded skin carcinoma (which is relatively rare in children) because these cases might not have been systematically registered (Goodwin *et al*, 2004), and non-CNS disease classified as non-malignant in previous standard coding systems. We assessed notifications received from the two principal sources of ascertainment to the NRCT: the CRs and the CCLG register (including its predecessor, the UKCCSG register). The two eligible cases ascertained only by death certificate were treated as CR notifications.

The NRCT had received all routine notifications for the study period by February 2007, when quarterly data exchange with the CRs began. Further information received by April 2009 included some notifications from CRs for previously CCLG-only cases. As these notifications could have been feedback of data originally received from the CCLG and notified to the CRs by the NRCT, analyses were performed under the extreme assumptions that either (A) all or (B) none were feedback. In both versions we took all other notifications as they were in April 2009.

#### Capture-recapture estimates of completeness

We used two-source capture-recapture to estimate the number of cases in the population, and hence the fraction registered (completeness). The nearly unbiased estimator of the number of cases in the population is defined as *a*+*b*+*c*+*((b* × *c)/(a*+*1))*, where *a* is the number of registrations notified both by the first and by the second source, *b* by the first source only, and *c* by the second source only, on the assumptions that, for each source, every case has the same probability of ascertainment, and that ascertainment of any case by one source is independent of ascertainment by the other (Hook and Regal, 1995).

We used stratification to adjust for variation in factors that might affect the probability of ascertainment. For children living in England, Wales and Scotland at diagnosis, we grouped the registrations by CR zone (the CR responsible for the address at which the child lived at the time of diagnosis, irrespective of whether the CR actually notified the case or not), by age at diagnosis (0, 1–4, 5–9 and 10–14 completed years), by the 12 main diagnostic groups defined by the first level of ICCC3 (Steliarova-Foucher *et al*, 2005), and by whether the child had died or not, as known to the NRCT in April 2009. For each factor, the stratified estimate of completeness was the overall number of registrations divided by the sum of the separate capture-recapture estimates for the number of cases in the population within each stratum.

For children living in England and Wales at diagnosis, we investigated the possibility that ascertainment might be less complete for patients of lower SES. From the Office for National Statistics we obtained the Carstairs deprivation index (Carstairs and Morris, 1989) for census wards in England and Wales in 2001 (Morgan and Baker, 2006). We grouped the census wards into quintile categories of the Carstairs index, each containing approximately the same number of children aged 0–14, using 2001 census population counts (Office for National Statistics, 2010a). We allocated each registration to the census ward that included the postcode of residence at diagnosis (Office for National Statistics, 2010b), and took the Carstairs quintile category number of that census ward as a measure of SES for the case (1=least deprived, 5=most deprived). We grouped the registrations as lymphoid leukaemia (ICCC3 site group I, subgroup a), leukaemia (ICCC3 site group I), CNS tumours (ICCC3 site group III), and non-CNS solid cancers except skin carcinomas (the remainder). We calculated a separate capture-recapture estimate for cases in each SES category within each diagnostic group. For lymphoid leukaemia, we repeated this analysis by age group.

#### Notification patterns

We used logistic regression models to investigate whether the proportion of cases notified by only one source varied with the factors used for stratification. We successively defined an ‘event’ as a registration being CR-only, or CCLG-only under feedback assumption (A), or CCLG-only under feedback assumption (B). For factors other than SES, we performed likelihood ratio tests for heterogeneity in the odds of an event, for strata in which there was at least one event. For SES, we performed likelihood ratio tests for trend in the odds of an event, within age-diagnosis groups containing at least 10 events. All tests were at the 5% significance level. Calculations were done in Stata version 11 (StataCorp, 2009).

#### Cross-check with Hospital Episode Statistics

The completeness estimates for childhood leukaemia were verified by a cross-check with HES. Leukaemia was chosen because (a) this is a large and relatively homogeneous group of diseases, (b) treatment usually involves many in-patient episodes, (c) the peak age at diagnosis is before the sixth birthday, and therefore within the follow-up time from birth spanned by the available HES data, and (d) completeness of ascertainment is of particular interest because of the positive association of recorded incidence with higher SES previously reported in NRCT data for lymphoid leukaemia in age group 1–4 years (Stiller *et al*, 2008).

The national HES database provided an electronic record of in-patient episodes since April 1997 at NHS-funded treatment centres in England for patients resident anywhere, or in Wales for residents of England only. No names were supplied, but each episode was marked with an identification number (ID) that had been defined automatically by HES from the available data. Various diagnosis codes were supplied for each episode, recording the clinical picture at the time of the episode, which was not necessarily a definitive diagnosis.

We assumed that all episodes with the same ID belonged to the same patient. However, we did not assume that all distinct IDs necessarily represented distinct patients, because it was possible that the identification data recorded for any individual patient might vary between episodes, due to change, error or omission.

We selected episodes that included a leukaemia diagnosis (ICD-10 C91 to C95) and that recorded the birth year as 1998. We grouped these episodes by ID, and selected IDs whose first leukaemia episode was before the end of 2004, so that any diagnosis made before the sixth birthday should be included. Where possible, the IDs were automatically matched with NRCT cases, by either the NHS number or the combination of date of birth, sex, and postcode. We manually checked the unmatched IDs. We also checked the matched NRCT case children who according to the NRCT had a non-leukaemia diagnosis, were not born in 1998, or were not resident in England at diagnosis.

## Results

### Notification patterns and capture-recapture estimates of completeness

Of the 2985 registrations for patients resident in England, Wales and Scotland at diagnosis, 206 (7%) were notified only by the CRs, and between 117 (4%) and 224 (8%) were notified only by the CCLG (Table 1). The number of CCLG-only notifications depended on the extent to which CR notifications received after February 2007 for previously CCLG-only cases were the result of feedback; the reported range represents the extreme possibilities that none (under assumption (B)) or all (under (A)) were feedback. The overall capture-recapture completeness estimate was 99% under assumption (A) and 100% under (B). Results stratified by CR zone, age group, diagnostic group, or whether the child had died, were very close to the crude estimate.

The percentage of CR-only notifications varied between registry zones (*P*<0.001), ranging from 2% in Mersey and the West Midlands to 22% in Wales. The percentage of CCLG-only notifications also varied between zones (*P*<0.001), and was highest in Eastern (10%) or Thames (6–18%), depending on the feedback assumption. The CCLG-only percentage was very low in Wales and the South West, West Midlands, and Oxford regions, suggesting that in these areas there may have been direct transfer of data from the local CCLG centre to the CR before the data were supplied to the NRCT. Excluding these four zones, and assuming feedback, the proportion notified by the CRs may have been as low as 1844 (90%) of 2054 registrations. The completeness estimates were 98–99% in Thames, 99% in Eastern, 99–100% in Scotland, and 100% elsewhere.

The percentage of CR-only notifications ranged from 4% in age group 1–4 to 11% in age group 10–14 years (*P*<0.001). Under feedback assumption (A), the CCLG-only percentage ranged from 6% in age group 10–14 years to 12% at age under 1 year (*P*=0.012). Under assumption (B), CCLG-only notification did not vary by age, the percentage being 3–5% in all age groups. The completeness estimate was 99–100% in age groups under 1 year and 10–14 years, and 100% at other ages.

CR-only notification varied between diagnostic groups (*P*<0.001), ranging from 0–3% for leukaemia, neuroblastoma, retinoblastoma and renal/hepatic cancers, to 14% for germ-cell/gonadal cancers, 39% for melanoma/carcinoma (i.e., malignant melanoma and non-skin carcinoma not included in other groups), and 78% for other/unspecified cancer (from only 23 registrations). Under feedback assumption (A), the percentage of CCLG-only notifications varied between diagnostic groups, ranging from 0% for hepatic cancer to 17% for retinoblastoma (*P*=0.036). Under assumption (B), the percentage of CCLG-only notifications did not significantly vary with diagnosis, the largest being 11% for retinoblastoma. The completeness estimate was 100% for leukaemia, neuroblastoma, retinoblastoma and renal/hepatic cancers, 99–100% for lymphoma, CNS tumours, bone cancer and soft-tissue sarcoma, 98–99% for germ-cell/gonadal cancer, 97–98% for melanoma/carcinoma, and 86% (from 23 registrations) for other/unspecified cancer.

The CR-only percentage was 5% for children who had died and 7% for other children (*P*=0.029). Under feedback assumptions (A) and (B), the CCLG-only percentage was respectively 2–5% for those who had died and 4–8% for others (*P*<0.001 and *P*=0.005). The completeness estimate was 100% for children who had died, and 99–100% for other children.

Among children resident in England and Wales at diagnosis, there was some variation with SES in notification patterns (Table 2). For CNS tumours, there was a decreasing trend with deprivation in CR-only notification (*P*=0.035); the percentage was 2% in the most deprived quintile, and 6–10% elsewhere. For leukaemia, under feedback assumption (A) only, there was an increasing trend with deprivation in CCLG-only notification (*P*=0.007); the percentage was 16% in the most deprived quintile, and 7–8% elsewhere. There was a similar pattern in the lymphoid subgroup (*P*=0.021, Table 2), which was not statistically significant when subdivided into age groups (Table 3). There was no evidence for trends under feedback assumption (B). For CNS tumours, leukaemia and lymphoid leukaemia, the completeness estimate was 100% in all five deprivation categories, under both feedback assumptions (Table 2); for lymphoid leukaemia by age group it was lower (98–99%) in the least deprived category for age group 10–14 only (Table 3). For non-CNS solid cancer, the completeness estimate was 99% in the most deprived category, and 99–100% elsewhere (Table 2).

### Cross-check with Hospital Episode Statistics

There were 432 different IDs in the HES file, of which 390 (90%) were automatically matched to 297 NRCT cases (on NHS number or the combination of date of birth, sex, and postcode), confirming that distinct IDs did not necessarily represent distinct patients. Of the 42 unmatched IDs, 31 had five or fewer leukaemia episodes, and 21 had only one. None of the episodes for these IDs recorded a death, and identifying information was often incomplete. Grouping by birthdate, sex, postcode and NHS number, as available, reduced the 42 unmatched IDs to 26 probably distinct patients. Comparing birthdate, sex, diagnosis, and episode dates, nine of these patients appeared to be possible matches for NRCT cases that had been linked to other IDs in the HES file, and four were definite matches for cases that were known to be ineligible for the NRCT. Two patients, with 37 and 44 episodes, respectively, were traced by enquiry at the relevant treatment centres, and were both found to be ineligible for the NRCT because they were not resident in the UK at diagnosis. Checking the full HES database, four patients (accounting for eight leukaemia episodes altogether) appeared to have a haematological but non-leukaemic diagnosis. The remaining seven patients could perhaps be explained as unidentified treatment fragments or errors, as they accounted for only 11 leukaemia episodes altogether.

Of the 297 NRCT cases that were matched to one or more of the 390 HES IDs, 269 were leukaemia patients who were born in 1998 and diagnosed before 2005 while resident in England, according to NRCT records. The remaining 28 matched NRCT cases did not fit these criteria for the following reasons: two had birth year 1997, not 1998; three were not residents of England at diagnosis; and 23 did not have a primary diagnosis of leukaemia (7 were non-cancer and 16 were non-leukaemia cancer). The NRCT contained four further relevant cases that were apparently not seen as NHS in-patients while resident in England, and were therefore correctly not in the HES file.

This cross-check appears to be consistent with a high level of completeness of ascertainment by the NRCT. Agreement with the relevant NRCT registrations would require that missing and inaccurate HES data accounted for the non-matching of some HES episodes and the matching of some inappropriate NRCT cases, as described. This does not seem implausible, given the demonstrated uncertainties of some of the identifying and diagnostic information in the HES records.

## Discussion

Capture-recapture suggests that the NRCT ascertained nearly all cases of childhood cancer diagnosed during 2003–04 in residents of Britain. Some of the completeness estimates were slightly reduced when it was assumed that all CR notifications received between February 2007 and April 2009 for previously CCLG-only cases were due to feedback through routine data exchange. Even under this assumption, however, the completeness estimate was 99% overall. Stratification by various factors that might affect the probability of ascertainment made almost no difference to the overall estimate.

The capture-recapture estimates must be interpreted with caution, because there was undoubtedly some dependence between the two sources. There may have been direct transfer of data to some individual CRs from their local CCLG clinical centres before the NRCT was notified. More generally, both sources use records from NHS cancer treatment centres, and both might tend to miss patients with non-fatal disease treated by clinicians not specialising in oncology.

Although the HES cross-check did not find convincing evidence of a serious failure of registration by the NRCT, we cannot be certain without further enquiries that no relevant cases were missed. Moreover, HES data are clearly not independent of other hospital-based sources of notification; in particular, the cross-check could not detect any unregistered children who were treated by non-NHS clinicians, or were diagnosed only around the time of death. Recent completeness of ascertainment for early-childhood leukaemia in residents of England may not be typical of other periods, ages and diagnostic groups, or of Britain in general.

It is clear that direct notification to the NRCT from specialist clinicians was a valuable supplement to the general cancer registration system. Of the 2985 patients known to the NRCT who were resident in Britain and diagnosed during 2003–2004, the CRs notified between 92% and 96% overall (depending on the feedback assumption), and no more than 90% in at least one region. Overall, the CCLG notified a similar percentage of registrations (93%).

During 1972–73 in the region of North West England covered by the Manchester Children's Tumour Registry (MCTR), 91% of known childhood cancer cases were ascertained to the MCTR from Hospital Activity Analysis records (a predecessor of HES), 93% from the regional CR, and 93% from clinicians; 98–99% could have been ascertained from any two of these three sources (Leck *et al*, 1976). The percentages notified by CR and clinicians seem remarkably similar to those reported here for national registration in Britain 30 years later.

A previous two-source capture-recapture study (Draper *et al*, 1991) estimated NRCT completeness to be 99% for childhood leukaemia and non-Hodgkin lymphoma in Britain diagnosed during 1966–83. Although the result resembles the estimates for leukaemia and lymphoma presented here, the assumption of independence was more questionable in the earlier study. In particular, the two sources of ascertainment were defined so that one source included notification from CRs whereas the other included notification from death certificates; during the 1960s and early 1970s, death certificates were an important component of non-CR ascertainment of leukaemia to the NRCT, and death certificates have always been a source of ascertainment to the CRs. Evidence from one region suggested a rather lower level of completeness during 1972–1984: a cross-check with clinical records found that 137 (95%) of 144 childhood leukaemia patients treated at hospitals in the Oxford region had been ascertained by the NRCT (Draper *et al*, 1989).

In New Zealand, for the diagnosis period 1990–93, three different sources of ascertainment notified, respectively, 97%, 98% and 86% of known cases of childhood cancer, with an overall capture-recapture estimate of nearly 100% (Dockerty *et al*, 1997). In three cities in Brazil, for childhood acute leukaemia diagnosed in 2001, there were 55, 44 and 28 known cases, respectively; overall, 48% were notified by CRs and 60% by a diagnostic laboratory database (Azevedo-Silva *et al*, 2009). Two-source capture-recapture estimates for the true numbers of cases in the three cities were 71, 82 and 89, implying relatively low completeness estimates of 77%, 54% and 31%, consistent with the difficulties of cancer registration in a huge and developing country.

In the present study, as far as possible, univariate stratified analysis was used to assess and allow for potential dependence between sources. This is equivalent to fitting a log-linear capture-recapture model with a single categorical covariate (Tilling *et al*, 2001). Analysis was also stratified by SES within diagnostic group and within age group. As there was very little evidence of variation in completeness with any of the factors considered, we chose not to apply the more sophisticated modelling methods that are appropriate for the joint effects of several categorical covariates, or for continuous covariates (Tilling and Sterne, 1999).

The proportion of registrations notified by the CCLG was very high (97% or more) for the typical cancers of infancy and early childhood normally diagnosed in specialist paediatric oncology centres (leukaemia, neuroblastoma, retinoblastoma, and renal and hepatic cancers), and rather lower in diagnostic groups that are relatively frequent in older children, teenagers and adults. Accordingly, the proportion notified by the CCLG was slightly lower at ages 10–14 years than in younger age groups. In contrast, the proportion notified by the CRs was lower for infants than for older children under one of the feedback assumptions (and may have been as low as 83% for retinoblastoma).

Both sources notified slightly higher proportions of registrations for patients who died than for other patients. This is consistent with the possibility that registration might be slightly more complete for patients with more aggressive cancers (because they are more likely to be seen by specialist clinicians) and for patients who die (because the death certificate is a source of notification to the CRs).

For leukaemia and lymphoid leukaemia registrations from England and Wales, under one of the two feedback assumptions only, the proportion of CCLG-only notifications increased with deprivation. There was a contrary trend in CR-only notification for CNS tumours, suggesting that in deprived communities a higher proportion of registered cases had been referred to CCLG centres. A slight deficit of CR notifications from deprived communities is a possible explanation for both types of trend.

As described, notification patterns varied slightly according to geographical area, diagnostic group, age at diagnosis, whether the patient had died, and (for CNS tumours and perhaps leukaemia) SES. However, effects on completeness were trivial, because the great majority of registrations were notified by both sources. The capture-recapture estimate was 99–100% in almost every subgroup; it was slightly lower in the Thames region (98–99%), and in two diagnostic groups that are relatively rare in children (germ-cell/gonadal cancer 98–99%, melanoma and non-skin carcinoma 97–98%). There was very little evidence that completeness varied according to age at diagnosis, or whether the patient had died. There was no evidence that ascertainment was appreciably less complete in deprived areas of England and Wales than in affluent areas.

## Conclusions

Of cases of childhood cancer in Britain diagnosed during 2003–04 and registered by the NRCT, 92–96% were notified by general cancer registries, and 93% by specialist clinicians. Assuming that these sources were independent, capture-recapture suggests that ascertainment was 99–100% complete. Stratification by various factors that might affect the probability of notification made no difference to the completeness estimate. As both sources used records from treatment centres, the independence assumption cannot be fully justified. With this caveat, ascertainment of recently diagnosed childhood cancer in Britain appears to be virtually complete.

## Figures and Tables

**Table 1:** Childhood cancer diagnosed during 2003–2004 in residents of England, Wales and Scotland

**Stratification factor**	**Number of registrations (% of total) by source of notification**				**Capture-recapture estimates**			
	**Total**	**CR only**	**CCLG only**		**Number of cases**		**Completeness**	
**CR feedback assumption:**			**A**	**B**	**A**	**B**	**A**	**B**
**Crude**	**2985**	**206 (7%)**	**224 (8%)**	**117 (4%)**	**3003.1**	**2994.1**	**99%**	**100%**
*CR zone of residence*		*P*(het)<0.001	*P*(het)<0.001	*P*(het)<0.001				
Eastern	293	24 (8%)	29 (10%)	28 (10%)	295.9	295.8	99%	99%
Mersey	123	2 (2%)	11 (9%)	10 (8%)	123.2	123.2	100%	100%
North west	218	11 (5%)	13 (6%)	9 (4%)	218.7	218.5	100%	100%
Northern/Yorkshire	325	10 (3%)	21 (6%)	11 (3%)	325.7	325.4	100%	100%
Oxford	157	8 (5%)	4 (3%)	1 (1%)	157.2	157.1	100%	100%
South West	333	24 (7%)	3 (1%)	2 (1%)	333.2	333.2	100%	100%
Thames	559	51 (9%)	103 (18%)	33 (6%)	571.9	562.5	98%	99%
Trent	261	14 (5%)	17 (7%)	10 (4%)	262.0	261.6	100%	100%
West Midlands	294	7 (2%)	7 (2%)	5 (2%)	294.2	294.1	100%	100%
Wales	147	32 (22%)	0 (0%)	0 (0%)	147.0	147.0	100%	100%
Scotland	275	23 (8%)	16 (6%)	8 (3%)	276.6	275.8	99%	100%
								
Stratified					3005.7	2994.0	99%	100%
								
*Age at diagnosis (years)*		*P*(het)<0.001	*P*(het)=0.012	*P*(het)=0.325				
Under 1	305	22 (7%)	38 (12%)	15 (5%)	308.4	306.2	99%	100%
1–4	995	38 (4%)	73 (7%)	38 (4%)	998.1	996.6	100%	100%
5–9	807	49 (6%)	56 (7%)	37 (5%)	810.9	809.5	100%	100%
10–14	878	97 (11%)	57 (6%)	27 (3%)	885.6	881.5	99%	100%
								
Stratified					3003.1	2993.8	99%	100%
								
*Diagnostic group*		*P*(het)<0.001	*P*(het)=0.036	*P*(het)=0.245				
Leukaemia	960	30 (3%)	77 (8%)	35 (4%)	962.7	961.2	100%	100%
Lymphoma	271	16 (6%)	22 (8%)	10 (4%)	272.5	271.7	99%	100%
CNS tumours	733	58 (8%)	41 (6%)	26 (4%)	736.7	735.3	99%	100%
Neuroblastoma	185	6 (3%)	17 (9%)	7 (4%)	185.6	185.2	100%	100%
Retinoblastoma	83	2 (2%)	14 (17%)	9 (11%)	83.4	83.3	100%	100%
Renal cancer	172	3 (2%)	14 (8%)	8 (5%)	172.3	172.2	100%	100%
Hepatic cancer	34	0 (0%)	0 (0%)	0 (0%)	34.0	34.0	100%	100%
Bone cancer	132	9 (7%)	11 (8%)	8 (6%)	132.9	132.6	99%	100%
Soft-tissue sarcoma	217	20 (9%)	11 (5%)	5 (2%)	218.2	217.5	99%	100%
Germ cell/gonadal	98	14 (14%)	12 (12%)	6 (6%)	100.3	99.1	98%	99%
Melanoma/carcinoma	77	30 (39%)	4 (5%)	2 (3%)	79.7	78.3	97%	98%
Other/unspecified cancer	23	18 (78%)	1 (4%)	1 (4%)	26.6	26.6	86%	86%
								
Stratified					3004.9	2996.9	99%	100%
								
*Known to have died?*		*P*(het)=0.029	*P*(het)<0.001	*P*(het)=0.005				
No	2332	173 (7%)	194 (8%)	103 (4%)	2349.1	2340.7	99%	100%
Yes	653	33 (5%)	30 (5%)	14 (2%)	654.7	653.8	100%	100%
								
Stratified					3003.8	2994.4	99%	100%

Abbreviations: A=all late CR notifications were feedback; B=none was; CCLG=Children's Cancer and Leukaemia Group; CNS=intracranial and intraspinal (central nervous system); CR=general cancer registry; *P*(het)=result of test for heterogeneity between categories.

**Table 2:** Childhood cancer diagnosed during 2003–2004 in residents of England and Wales

**Diagnostic group Deprivation category**	**Number of registrations (% of total) by source of notification**				**Capture-recapture estimates**			
	**Total**	**CR only**	**CCLG only**		**Number of cases**		**Completeness**	
**CR feedback assumption:**			**A**	**B**	**A**	**B**	**A**	**B**
*Lymphoid leukaemia*		*P*(trend)=0.341	*P*(trend)=0.021	*P*(trend)=0.566				
1=least deprived	141	4 (3%)	11 (8%)	7 (5%)	141.4	141.2	100%	100%
2	150	2 (1%)	12 (8%)	7 (5%)	150.2	150.1	100%	100%
3	128	1 (1%)	8 (6%)	2 (2%)	128.1	128.0	100%	100%
4	117	1 (1%)	12 (10%)	3 (3%)	117.1	117.0	100%	100%
5=most deprived	131	2 (2%)	21 (16%)	10 (8%)	131.4	131.2	100%	100%
								
Stratified					668.1	667.5	100%	100%
Crude	667	10 (1%)	64 (10%)	29 (4%)	668.1	667.5	100%	100%
								
*Leukaemia*		*P*(trend)=0.064	*P*(trend)=0.007	*P*(trend)=0.487				
1=least deprived	182	10 (5%)	13 (7%)	8 (4%)	182.8	182.5	100%	100%
2	193	5 (3%)	13 (7%)	8 (4%)	193.4	193.2	100%	100%
3	168	4 (2%)	12 (7%)	4 (2%)	168.3	168.1	100%	100%
4	158	4 (3%)	13 (8%)	3 (2%)	158.4	158.1	100%	100%
5=most deprived	166	3 (2%)	26 (16%)	12 (7%)	166.6	166.2	100%	100%
								
Stratified					869.4	868.1	100%	100%
Crude	867	26 (3%)	77 (9%)	35 (4%)	869.6	868.1	100%	100%
								
*CNS tumours*		*P*(trend)=0.035	*P*(trend)=0.206	*P*(trend)=0.176				
1=least deprived	134	12 (9%)	6 (4%)	5 (4%)	134.6	134.5	100%	100%
2	147	14 (10%)	6 (4%)	3 (2%)	147.7	147.3	100%	100%
3	141	9 (6%)	3 (2%)	3 (2%)	141.2	141.2	100%	100%
4	133	11 (8%)	5 (4%)	4 (3%)	133.5	133.4	100%	100%
5=most deprived	115	2 (2%)	10 (9%)	8 (7%)	115.2	115.2	100%	100%
								
Stratified					672.1	671.6	100%	100%
Crude	670	48 (7%)	30 (4%)	23 (3%)	672.4	671.8	100%	100%
								
*Non-CNS solid cancer*		*P*(trend)=0.858	*P*(trend)=0.072	*P*(trend)=0.277				
1=least deprived	242	18 (7%)	19 (8%)	12 (5%)	243.7	243.0	99%	100%
2	213	26 (12%)	12 (6%)	6 (3%)	214.8	213.9	99%	100%
3	220	17 (8%)	21 (10%)	9 (4%)	222.0	220.8	99%	100%
4	250	32 (13%)	19 (8%)	5 (2%)	253.0	250.8	99%	100%
5=most deprived	248	16 (6%)	30 (12%)	19 (8%)	250.4	249.4	99%	99%
								
Stratified					1183.8	1177.8	99%	100%
Crude	1173	109 (9%)	101 (9%)	51 (4%)	1184.4	1178.5	99%	100%

Abbreviations: A=all late CR notifications were feedback; B=none was; CCLG=Children's Cancer and Leukaemia Group; CNS=intracranial and intraspinal (central nervous system); CR=general cancer registry; *P*(trend)=result of test for trend over categories.

**Table 3:** Childhood lymphoid leukaemia diagnosed during 2003–2004 in residents of England and Wales

**Age at diagnosis Deprivation category**	**Number of registrations (% of total) by source of notification**				**Capture-recapture estimates**			
	**Total**	**CR only**	**CCLG only**		**Number of cases**		**Completeness**	
**CR feedback assumption:**			**A**	**B**	**A**	**B**	**A**	**B**
*Age under 1 year*		—	—	—				
1=least deprived	5	0 (0%)	0 (0%)	0 (0%)	5.0	5.0	100%	100%
2	6	1 (17%)	0 (0%)	0 (0%)	6.0	6.0	100%	100%
3	4	0 (0%)	0 (0%)	0 (0%)	4.0	4.0	100%	100%
4	11	0 (0%)	3 (27%)	0 (0%)	11.0	11.0	100%	100%
5=most deprived	5	1 (20%)	0 (0%)	0 (0%)	5.0	5.0	100%	100%
								
Stratified					31.0	31.0	100%	100%
Crude	31	2 (6%)	3 (10%)	0 (0%)	31.2	31.0	99%	100%
								
*Age 1–4 years*		—	*P*(trend)=0.068	*P*(trend)=0.674				
1=least deprived	62	2 (3%)	5 (8%)	5 (8%)	62.2	62.2	100%	100%
2	78	1 (1%)	6 (8%)	4 (5%)	78.1	78.1	100%	100%
3	67	1 (1%)	4 (6%)	0 (0%)	67.1	67.0	100%	100%
4	57	1 (2%)	7 (12%)	2 (4%)	57.1	57.0	100%	100%
5=most deprived	76	1 (1%)	12 (16%)	5 (7%)	76.2	76.1	100%	100%
								
Stratified					340.7	340.3	100%	100%
Crude	340	6 (2%)	34 (10%)	16 (5%)	340.7	340.3	100%	100%
								
*Age 5–9 years*		—	*P*(trend)=0.658	-				
1=least deprived	49	0 (0%)	2 (4%)	0 (0%)	49.0	49.0	100%	100%
2	39	0 (0%)	4 (10%)	2 (5%)	39.0	39.0	100%	100%
3	38	0 (0%)	2 (5%)	1 (3%)	38.0	38.0	100%	100%
4	31	0 (0%)	1 (3%)	1 (3%)	31.0	31.0	100%	100%
5=most deprived	29	0 (0%)	3 (10%)	2 (7%)	29.0	29.0	100%	100%
								
Stratified					186.0	186.0	100%	100%
Crude	186	0 (0%)	12 (6%)	6 (3%)	186.0	186.0	100%	100%
								
*Age 10–14 years*		—	*P*(trend)=0.305	—				
1=least deprived	25	2 (8%)	4 (16%)	2 (8%)	25.4	25.2	98%	99%
2	27	0 (0%)	2 (7%)	1 (4%)	27.0	27.0	100%	100%
3	19	0 (0%)	2 (11%)	1 (5%)	19.0	19.0	100%	100%
4	18	0 (0%)	1 (6%)	0 (0%)	18.0	18.0	100%	100%
5=most deprived	21	0 (0%)	6 (29%)	3 (14%)	21.0	21.0	100%	100%
								
Stratified					110.4	110.2	100%	100%
Crude	110	2 (2%)	15 (14%)	7 (6%)	110.3	110.1	100%	100%

Abbreviations: A=all late CR notifications were feedback; B=none was; CCLG=Children's Cancer and Leukaemia Group; CR=general cancer registry; *P*(trend)=result of test for trend over deprivation categories, for age groups with at least 10 relevant notifications.

## References

[bib1] Adelman AS, Groves FD, O'Rourke K, Sinha D, Hulsey TC, Lawson AB, Wartenberg D, Hoel DG (2007) Residential mobility and risk of childhood acute lymphoblastic leukaemia: an ecological study. Br J Cancer 97: 140–144 17533404 10.1038/sj.bjc.6603793 PMC2359674

[bib2] Azevedo-Silva F, Reis RdS, Santos MdO, Luiz RR, Pombo-de-Oliveira MS (2009) Evaluation of childhood acute leukemia incidence and underreporting in Brazil by capture-recapture methodology. Cancer Epidemiol 33(6): 403–405 19833572 10.1016/j.canep.2009.09.004

[bib3] Carstairs V, Morris R (1989) Deprivation, mortality and resource allocation. Community Med 11(4): 364–372 2634516

[bib4] Dockerty JD, Becroft DMO, Lewis ME, Williams SM (1997) The accuracy and completeness of childhood cancer registration in New Zealand. Cancer Causes Control 8(6): 857–864 9427428 10.1023/a:1018412311997

[bib5] Draper GJ, Bower BD, Darby SC, Doll R (1989) Completeness of registration of childhood leukaemia near nuclear installations and elsewhere in the Oxford region. BMJ 299(6705): 952 2508950 10.1136/bmj.299.6705.952 PMC1837824

[bib6] Draper GJ, Stiller CA, O'Connor CM, Vincent TJ, Elliott P, McGale P, Rodrigues L, Hills M, Black RJ, Sharp L, Urquhart JD, Alexander FE, Gilman EA, Knox EG, Besag J, Newell J, Craft A, Openshaw S, Cuzick J, Gardner MJ (1991) The Geographical Epidemiology of Childhood Leukaemia and Non-Hodgkin Lymphomas in Great Britain, 1966–83, OPCS Studies on Medical and Population Subjects No.53. OPCS: London

[bib7] Goodwin RG, Holme SA, Roberts DL (2004) Variations in registration of skin cancer in the United Kingdom. Clin Exp Dermatol 29(3): 328–330 15115532 10.1111/j.1365-2230.2004.01523.x

[bib8] Hook EB, Regal RR (1995) Capture-recapture methods in epidemiology: methods and limitations. Epidemiol Rev 17(2): 243–264 8654510 10.1093/oxfordjournals.epirev.a036192

[bib9] Leck I, Birch JM, Marsden HB, Steward JK (1976) Methods of classifying and ascertaining children′s tumours. Br J Cancer 34(1): 69–82 952716 10.1038/bjc.1976.124 PMC2025137

[bib10] Morgan O, Baker A (2006) Measuring deprivation in England and Wales using 2001 Carstairs scores. Health Stat Q 31: 28–33 16972693

[bib11] Office for National Statistics 2001. Census: Standard Area Statistics (England and Wales). ESRC/JISC Census Programme. (2010a) Census Dissemination Unit, Mimas (University of Manchester)

[bib12] Office for National Statistics Postcode Directories. ESRC Census Programme. (2010b) Census Dissemination Unit, Mimas (University of Manchester)

[bib13] Parkin DM, Bray F (2009) Evaluation of data quality in the cancer registry: Principles and methods Part II. Completeness. Eur J Cancer 45(5): 756–764 19128954 10.1016/j.ejca.2008.11.033

[bib14] Poole C, Greenland S, Luetters C, Kelsey JL, Mezei G (2006) Socioeconomic status and childhood leukaemia: a review. Int J Epidemiol 35(2): 370–384 16308412 10.1093/ije/dyi248

[bib15] Quinn M, Babb P, Brock A, Kirby L, Jones J (2001) Cancer Trends in England and Wales 1950–1999. Office for National Statistics: London

[bib16] StataCorp (2009) Stata Statistical Software: Release 11. StataCorp LP: College Station, TX

[bib17] Steliarova-Foucher E, Stiller C, Lacour B, Kaatsch P (2005) International classification of childhood cancer, third edition. Cancer 103(7): 1457–1467 15712273 10.1002/cncr.20910

[bib18] Stiller CA, Kroll ME, Boyle PJ, Feng Z (2008) Population mixing, socioeconomic status and incidence of childhood acute lymphoblastic leukaemia in England and Wales: analysis by census ward. Br J Cancer 98(5): 1006–1011 18253115 10.1038/sj.bjc.6604237 PMC2266854

[bib19] Tilling K, Sterne JA (1999) Capture-recapture models including covariate effects. Am J Epidemiol 149(4): 392–400 10025483 10.1093/oxfordjournals.aje.a009825

[bib20] Tilling K, Sterne JA, Wolfe CD (2001) Estimation of the incidence of stroke using a capture-recapture model including covariates. Int J Epidemiol 30(6): 1351–1359 11821345 10.1093/ije/30.6.1351

